# A case of paraplegia due to asymptomatic varicella-zoster virus infection in AIDS patient unexpectedly diagnosed by CSF metagenomic next-generation sequencing

**DOI:** 10.1186/s12879-021-06611-9

**Published:** 2021-09-16

**Authors:** Zhiman Xie, Jingzhen Lai, Chuanyi Ning, Guangjing Ruan, Hao Liang

**Affiliations:** 1 Infectious Disease Department, No. 4th People’s Hospital of Nanning and the Affiliated Nanning Infectious Diseases Hospital of Guangxi Medical University, Guangxi Medical University, No. 1, Second Lane, Changgang Road, Nanning, 530023 Guangxi China; 2 Guangxi Key Laboratory of AIDS Prevention and Treatment & Guangxi Universities Key Laboratory of Prevention and Control of Highly Prevalent Disease, School of Public Health, Guangxi Medical University, No. 22 Shuangyong Road, Nanning, 530021 Guangxi China; 3 Guangxi Collaborative Innovation Center for Biomedicine, Life Sciences Institute, Guangxi Medical University, No. 22 Shuangyong Road, 530021 Nanning, Guangxi China; 4 Nursing College, Guangxi Medical University, No. 8 Shuangyong Road, Nanning, 530021 Guangxi China

**Keywords:** HIV, AIDS, Varicella-zoster virus, Paraplegia, mNGS

## Abstract

**Background:**

Varicella-zoster virus (VZV) infection may induce central nervous system complications in HIV/AIDS patients. However, it is rare to have paraplegia caused by VZV infection but no herpes zoster clinically. Asymptomatic VZV infection in HIV/AIDS patient increased the difficulty of diagnosis.

**Case presentation:**

We reported a 41-year-old male AIDS patient with rare asymptomatic VZV infection-induced paraplegia after his anti-retroviral therapy initiation. MRI of the spinal cord showed the morphology of the thoracic spinal cord was irregular and locally inflated. The patient was confirmed as VZV induced thoracic myelomyelitis by using the cerebrospinal fluid for metagenomic next-generation sequencing (mNGS).

**Conclusions:**

mNGS may contribute to disease diagnosis for asymptomatic VZV infection-induced myelitis.

**Supplementary Information:**

The online version contains supplementary material available at 10.1186/s12879-021-06611-9.

## Background

Varicella-zoster virus (VZV) is also known as Human_alphaherpesvirus_3. VZV infection can cause primary varicella infection (chickenpox) in children, meanwhile a few cases will develop to herpes zoster in adults after reactivation [1]. The incidence of herpes zoster in anti-retroviral therapy (ART)-naïve human immunodeficiency virus (HIV) infected patients can reach 9.4 % [2]. In HIV/AIDS patients, the risk of VZV infection increased and induced many related complications, including central nervous system (CNS) complications [3]. However, it is rare to have paraplegia caused by direct invasion of the spinal cord by VZV but no herpes zoster clinically in HIV-VZV coinfected patients. Here we present a rare case of myelitis due to VZV infection without VZV-related clinical symptoms in AIDS patients who were diagnosed by cerebrospinal fluid (CSF) MAPMI™, a metagenomic next-generation sequencing technology (mNGS).

## Case presentation

A 41-year-old male was presented in our hospital on February 15th, 2020, for bilateral lower limb paralysis for more than 10 days. In the middle of January 2020, he was admitted in the local hospital due to he had fever for more than one month. He was diagnosed with AIDS, bacterial pneumonia, and cytomegalovirus (CMV) infection in the local hospital. Seven hundreds copies /ml of CMV were detected in urine On January 16th, and 5.77 × 10^3^ copies /ml of CMV were detected in sputum on January 20th. After antimicrobial and anti-CMV treatment, the patient’s condition improved. The combined ART regimen of Nevirapine, Lamivudine, and Tenofovir was taken on January 27th, 2020. In the following days after cART initiation, he experienced weakness of lower limbs and developed paralysis of both legs and incontinence in a short time. He reported no skin lesions, fear of cold, fever, dizziness, headache, vomiting, limb convulsions, abdominal pain, diarrhea, or any other discomfort. He also reported no special medical history or trauma history. We gave him a physical examination when admission: his lower limb muscle tension was low, and the muscle strength was level 0. But the passive range of motion of all joints of the body was normal. His sensation disappeared in the sacrococcyx, buttocks, front and lateral thighs, back and medial sides, calves, and feet. However, pathological emission did not elicit.

**Fig. 1:**
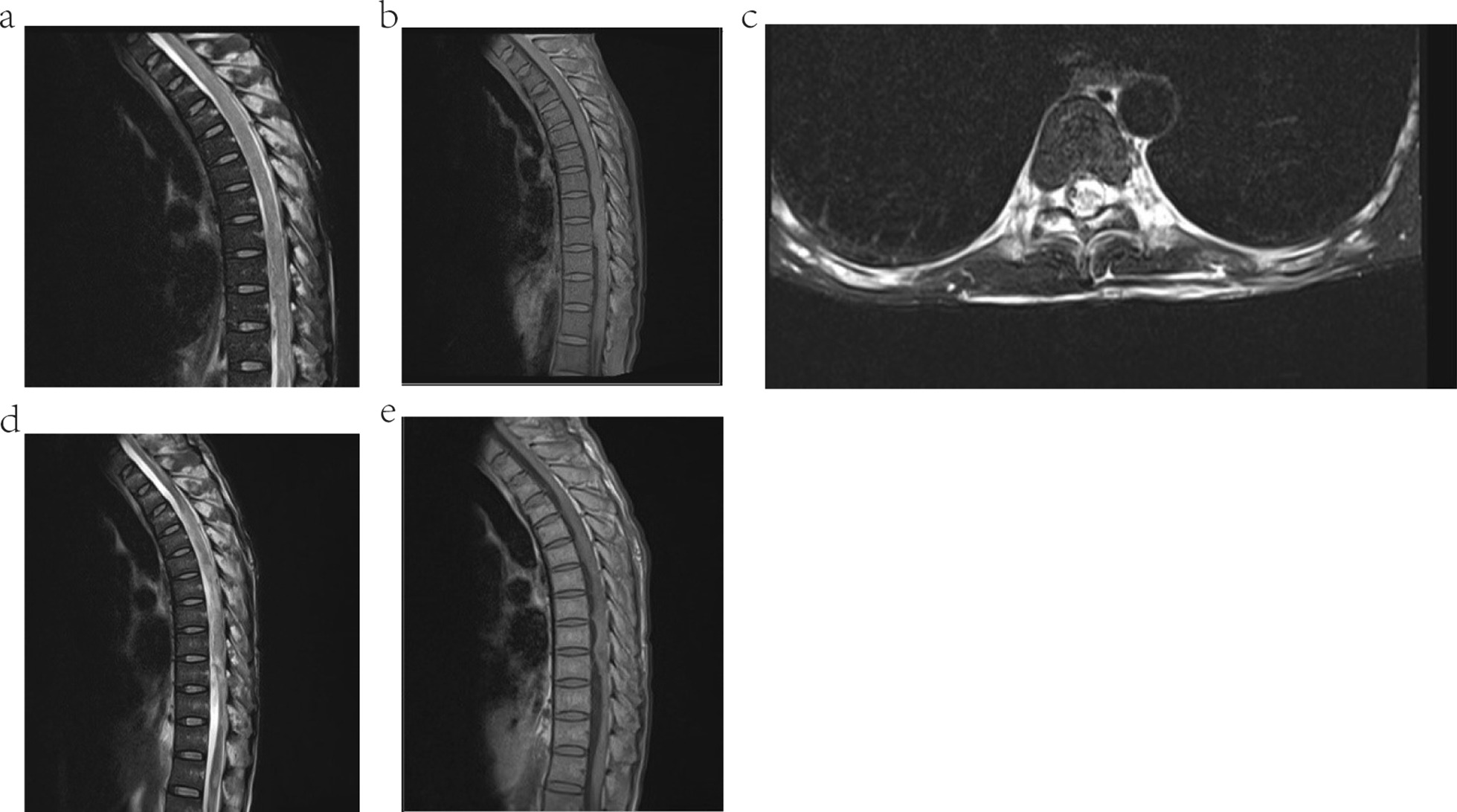
Spinal cord MRI and the mNGS result of the patient. **a** and **d** were the spinal cord segmental MRI T2-weighted image of the patient, **b** and **e** were the spinal cord segmental MRI T1-weighted image of the patient, **c** was the axial spinal cord segmental MRI T2-weighted image of the patient. **a**–**c** were recorded on February 17th, 2020. **a**–**c** showed that the thoracic spinal cord was irregular in shape with local enlargement and spotted and patchy hypersignal, which indicated edema or infection. **d**, **e** were recorded on April 3rd, 2020. Compared to **a**, **b** the edema spinal cord was reduced after treatment

The clinical tests after admission showed that the CD4^+^ T cell count was 35 cells/µL, white blood cells (WBC) count was 4.8 × 10^9^/L, red blood cell (RBC) count was 3.24 × 10^12^/L, hemoglobin (Hb) was 90 g/L, Platelets (PLT) was 259 × 10^9^/L, and the percentage of neutrophile granulocyte was 76.4 %. No abnormalities were found in liver and renal function tests. Screening for hepatitis B virus infection, hepatitis C virus infection, and syphilis were negative. Neither was the Mycobacterium tuberculosis (Mtb) infecting T cells test. DNA tests for CMV and Mtb in blood and CSF were negative, too. CSF examination showed the opening pressure was 60 mm H_2_O by lumbar puncture, the Pandy test was weakly positive, 16.00 × 10^6^ WBC/L, and a normal level of adenosine deaminase (2.01 U/L). The biochemical indicators of CSF revealed protein concentration was 960.60 mg/L (normal range, 150–450 mg/L), normal level of glucose (3.59 mmol/L), and chloride concentration was 111.30 mmol/L (normal range, 120–132 mmol/L). No bacteria or fungi was found in CSF smear and culture. MRI of the brain and electrocardiography were normal. However, MRI of the spinal cord showed the morphology of the thoracic spinal cord was irregular and locally inflated, and the signals of T1 and T2 were slightly longer (Fig. 1a–c). It indicated infection.

To figure out the pathogens that induced the infection in the spinal cord, the CSF was used for the super broad-spectrum pathogenic microorganisms mNGS by MAPMITM (CapitalBio MedLab Co. LTD, Beijing, China). The mNGS was done on BioelectronSeq4000 platform (CapitalBio). After quality filtering, eliminating replicate reads, removing reads matching human genome sequence, reads were classified against the NCBI reference/representative bacterial, archaeal, and fungal genomes, as well as genomes of DNA viruses infecting humans and DNA bacteriophages. Of 25,337,336 quality filtered sequencing reads, 23,485,313 were human, 53,502 were assigned to microbe, and 3935 were mapped on Human_alphaherpesvirus_3 (VZV). The VZV genome coverage was 95.55 %, and the estimated VZV DNA concentration was 2.6 × 10^3^copies/ml (computed from VZV to human read counts ratio combined with a TaqMan-based quantification of human DNA). Screening results for bacteria, fungi, and parasites were negative. The results indicated the pathogen was VZV (three representative DNA sequences got from mNGS and the reference sequence were showed in Additional file 1 and Additional file 2). The patient was confirmed as AIDS with VZV induced thoracic myelomyelitis.

After admission, he was treated with sodium phosphonate 3.0 g/3 times a day for 50 days, adjuvanted with traditional Chinese acupuncture for more than a month to control the symptom. Acyclovir 0.5 g/3 times a day was used for 22 days after he was diagnosis as VZV infection by mNGS. The MRI reexamination of the spinal on April 3rd showed that the edema of the lesion was reduce (Fig. 1d, e). However, his disease progression did not improve or get worse after more than 1 month’s treatment. Therefore, he abandoned further treatment automatically and was discharged from our hospital.

## Discussion and conclusions

VZV is an enveloped DNA virus, which can invade tissues of ectodermal origin, including skin, mucous membranes, and nerve tissue. Typical VZV-related manifestations include rash and pain [4]. VZV infection can cause a wide range of neurological diseases, such as postherpetic neuralgia, facial paralysis with Ramsay-Hunt syndrome, meningitis, encephalitis, myelitis, encephalomyelitis, and cerebrovascular disease, etc. [5–8].

The risk of VZV infection in HIV/AIDS patients was 2.5-fold higher compared with the general population [9]. It was estimated that subclinical reactivation of VZV would be found in 15 % of HIV-positive subjects, most of whom were asymptomatic [10]. The higher incidence of zoster in HIV/AIDS patients was reported to be associated with lower CD4 counts [11]. Additionally, ART initiation was reported to be associated with zoster occurrence in HIV/AIDS patients [12]. VZV meningitis may be accompanied with or without shingles, but VZV myelitis without a rash is rare. It is even rarer that VZV myelitis induced paraplegia without rash. The diagnosis of VZV myelitis is mostly based on the time correlation between typical skin lesions and myelopathy. MRI examination is helpful for diagnosis and differential diagnosis. However, it is difficult to diagnose VZV myelitis clinically without a rash. For VZV myelitis induced paraplegia patient without rash, the lack of an effective direct detection method for virus in CSF may result in poor prognosis or death. It was difficult to isolate the virus in the blood and CSF of patients, so VZV infection could be diagnosed through typical rash, clinical symptoms of spinal cord injury and MRI.

The clinical manifestations of the reported case didn’t include chills and fever, dizziness, headache, abdominal pain, diarrhea, or herpes zoster. Paraplegia is rarely the only manifestation of VZV infection. Therefore, the diagnosis cannot be made based on the manifestations. Here, we have made a pioneering attempt to isolate the pathogen DNA in CSF for mNGS. mNGS a high-throughput sequencing technology, has been applicated in clinical diagnosis for some unclear pathogens. With the application of mNGS, we found the sequences of VZV with an estimated viral load of 2.6 × 10^3^copies/mL, and no other viruses, bacteria, fungi, or other pathogens were found. The sequencing result confirmed the diagnosis of the patient, backing by his symptoms and clinical tests. We suggest that a routine test for VZV DNA in CSF should be established for the HIV-infected patients with neurological diseases, such as PCR for VZV DNA.

The patient was treated with sodium phosphonate 3.0 g/3 times a day and acyclovir 0.5 g/3 times a day during hospitalization. Acyclovir intravenous treatment is a recommended regimen for VZV infection [13]. Early antiviral and steroid therapy are the most important factor in the good prognosis of such patients. But steroid was not given in this case, because its side effects and it may mask other symptoms of unidentified opportunistic infections. In addition, it could be that intravenous acyclovir interferes with the rash, which leads to no rash manifestation in this patient.

Here, we reported a rare case of myelitis paraplegia caused by VZV without herpes-related symptoms, and share examples of metagenomic sequencing used in clinical diagnosis of difficult diseases, to provide a reference for other similar diseases.

## Supplementary Information

**Additional file 1**. Three representative DNA sequences got from CSF by mNGS.

**Additional file 2**. The reference sequence for sequencing analysis.

## Data Availability

The datasets generated and/or analysed during the current study are not publicly available due to ethical and legal reasons, but are available from the corresponding author on reasonable request.

## Abbreviations

AIDS: Acquired Immune Deficiency Syndrome
cART: Combined Anti-Retroviral Therapy
CMV: Cytomegalovirus
CNS: Central Nervous System
CSF: Cerebrospinal Fluid
DNA: Deoxyribonucleic Acid
Hb: Hemoglobin
HIV: Human Immunodeficiency Virus
mNGS: metagenomic Next-Generation Sequencing
MRI: Magnetic Resonance Imaging
PLT: Platelets
RBC: Red Blood Cell
VZV: Varicella-Zoster Virus
WBC: White Blood Cells
